# Phloxine B-loaded polymersomes enable eradication of *Pseudomonas aeruginosa* and *Staphylococcus aureus* in antimicrobial photodynamic therapy†

**DOI:** 10.1039/d5ra02238j

**Published:** 2025-06-04

**Authors:** Nicola Cusick, Holger Schönherr

**Affiliations:** a Physical Chemistry I & Research Center of Micro- and Nanochemistry and (Bio)Technology (Cμ), Department of Chemistry and Biology, School of Science and Technology, University of Siegen 57076 Siegen Germany schoenherr@chemie.uni-siegen.de

## Abstract

Utilised for decades in cancer therapy, the application of photosensitisers in antimicrobial photodynamic therapy is now well explored. In a smart wound dressing, an externally triggered antimicrobial strategy would enable on-demand infection eradication. Hence, in this work, the light-initiated production of reactive oxygen species from photosensitiser-loaded nanocarriers was explored. Such systems enable highly localised delivery of photosensitiser, without associated “dark toxicity” effects. The amphiphilic block copolymer PEG-*block*-PLA was synthesised *via* ring-opening polymerisation in the melt. *Via* the solvent shift method, the polymer was self-assembled into nanosized vesicles encapsulating Phloxine B, a commercial water-soluble photosensitiser. *In vitro* bacteria experiments with Phloxine B-loaded vesicles relied on localised illumination with green light (530 nm, 4 mW cm^−2^, 15 minutes) to generate ^1^O_2_, killing the bacterial cells. A 6.8 log_10_ reduction in CFU mL^−1^ for *Staphylococcus aureus* and a 4.7 log_10_ reduction in CFU mL^−1^ for *Pseudomonas aeruginosa* are reported. Incorporation of these vesicles into a support matrix in combination with a reporter dye could provide a pathway towards promising smart wound dressings.

## Abstract 2

Phloxine B is loaded into PEG-*b*-PLA vesicles which, upon green light irradiation, eradicate *Pseudomonas aeruginosa* and *Staphylococcus aureus*.

## Graphical abstract

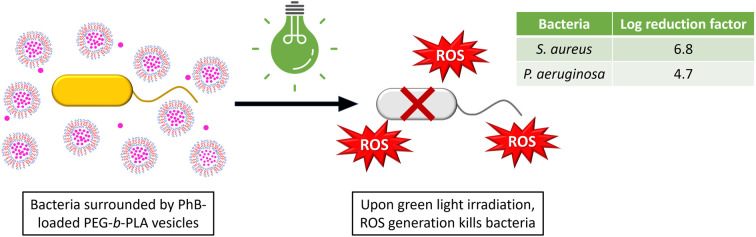

## 1. Introduction

In developed countries, an estimated 1–2% of the population will experience a chronic wound in their lifetime.^1,2^ A chronic wound can be defined as an open wound which has not progressed through the normal healing process, with an increased risk of infection.^2^ The two most prevalent pathogens present in chronic wound infections are *Pseudomonas aeruginosa* and *Staphylococcus aureus.*^3^ These bacteria are also found as lead pathogens in burn wounds.^4^ Typical therapies combine wound management strategies such as debridement with antibiotic administration.^5^ However, an inadequate dosage can promote the development of antibiotic resistance; 70% of wound infection-causing bacteria are resistant to at least one of the most common antibiotics.^6–8^ Infections caused by such antibiotic resistant pathogens are more difficult to treat due to limited effective therapeutics.^9^ Hence, new therapeutics less likely to lead to the development of antimicrobial resistance, as well as those able to treat resistant pathogens, are urgently required.^4^ Prime examples include antimicrobial peptides, which act as broad-spectrum membrane permeabilizers.^5,10^

Antimicrobial resistance to light-based approaches, such as visible light activation of photosensitisers (PSs), or the use of UVC light, are rarely reported.^6,11,12^ Antimicrobial photodynamic therapy (aPDT) using PSs therefore represents a viable alternative strategy to traditional antibiotics for the treatment of infected wounds. aPDT requires a PS, molecular oxygen, and light of an appropriate wavelength.^11^ The absorption of light by the PS followed by intersystem crossing results in the formation of the PS triplet excited state, which can interact with molecular oxygen to produce reactive oxygen species (ROS) *via* electron transfer (Type I) or energy transfer (Type II).^13^ These ROS cause oxidative stress to cells, hence PSs act as broad-spectrum antibacterial and antifungal killing agents.^14^ PDT is already clinically approved for multiple conditions, including a variety of cancers.^15^ The first clinically used PS, hematoporphyrin, was first used in the 1970s in the United States.^3^ Limitations of these first generation PSs, including short wavelength absorption and prolonged patient photosensitivity, lead to research into so-called second generation PSs with longer wavelength absorption.^16^ Such PSs are largely based on porphyrin, with absorption wavelengths >600 nm.^17^

The use of PDT to eradicate bacteria, aPDT, has received growing research interest in the previous two decades.^18^ It is particularly advantageous as it can provide localised treatment to fight infections caused by both Gram-positive and Gram-negative pathogens, as well as fungal and viral infections.^8^ A wide variety of PSs are available, with different chemical properties, degrees of hydrophilicity, and absorption maxima.^19^ A suitable PS can therefore be selected for a specific application. Halogenated xanthenes such as Rose Bengal possess large singlet oxygen quantum yields and are photoactivated in the visible light region.^20^ This is advantageous for superficial infection treatment, where light is not required to penetrate through tissue.^21^ However, as the diffusion of singlet oxygen is limited to a few hundred nanometres (estimations of ∼268 nm over 2 lifetimes in a cell have been reported),^22^ and it has a short half-life of ∼4 × 10^−6^ s in H_2_O, it is only cytotoxic to cells in the immediate vicinity.^23,24^ Therefore, the efficacy of aPDT depends on the localisation, and approaches in which photosensitisers are encapsulated or conjugated with targeting moieties are reported to enhance PS delivery by more accurate targeting to cells, but also by preventing PS aggregation in solution.^7,25^ These third generation photosensitisers are reported to overcome limitations of neat PS administration such as limited solubility, uncontrolled PS release, photo-bleaching and poor selectivity.^9^

Many PSs are inherently “theranostic” (therapeutic and diagnostic) as their fluorescence can be utilised in detection of pathogens.^26^ For the application of PSs in a wound dressing, it is likely that the PS would be located at the wound bed for a period of time and only irradiated upon the presence of infection. Hence, encapsulation is important to prevent contact of the PS with the initial small population of bacteria of a non-infected wound, which would provide time for bacteria to adapt and develop resistance.^8^ Moreover, diffusion of a PS-loaded carrier is much slower than the PS itself, providing a higher local concentration. It has been reported that aPDT nanosystems enable improved PS solubility, a reduction in the required PS concentration, controlled release, increased penetration and selectivity.^9,27^ For example, lipid nanoparticles encapsulating the PS toluidine blue O resulted in increased PS uptake by *S. aureus* (ATCC 6538), *P. aeruginosa* (ATCC 27853) and *E. coli* (ATCC 25922), more sustained ^1^O_2_ generation and improved bacterial inactivation due to improvements in solubility.^25^ However, CFU reductions were no greater than 4 log_10_.^22^ To improve stability, versatility and robustness of such systems, polymer-based nanocarriers have also been explored for delivery of PSs.^28,29^ Amphiphilic block copolymers, in which a hydrophilic and hydrophobic chain are covalently bonded, provide a facile approach to self-assemble kinetically trapped nanostructures.^30^ These structures are reported to be more stable, mechanically stronger and less permeable to small water-soluble molecules than liposomes.^31^

Often, the biocompatible polymer poly(ethylene glycol) (PEG) is utilised as the hydrophilic block as it is hypothesised to provide a stealth function, prolonging circulation times by resisting protein adsorption.^32,33^ As previously reported, the use of polyesters such as poly(caprolactone) (PCL) or poly(lactic acid) (PLA) as the hydrophobic block can provide an enzyme-responsive function to the vesicles; the ester bonds are cleaved by proteases and lipases.^34,35^ A biodegradable polymersome composed of polyester-containing block copolymers can be degraded in the biological environment in the presence of esterases, releasing the cargo molecules *via* simple ester hydrolysis.^32^ For example, the lipophilic PS hypocrellin A (HA) was loaded into PEG-*b*-PCL micelles and the lipase-dependent release of HA in the presence of MRSA was observed.^33^ Irradiation (*λ* = 470 nm, 90 mW cm^−2^, 60 minutes, 324 J cm^−2^) afforded complete killing of the pathogen, but had a higher minimum inhibitory concentration compared to neat HA *in vitro*.^34^

Previous work in our group highlighted the encapsulation of the hydrophobic photosensitiser Ru(Phen)_3_ in PEG-*b*-PLA micelles and vesicles.^36^ A reduction of at least 4.7 log_10_ in CFU mL^−1^ was reported against *P. aeruginosa* upon irradiation (*λ* = 455 nm, 3 mW cm^−2^, 30 minutes, 5.4 J cm^−2^) of vesicle suspensions with high Ru(Phen)_3_ concentrations (187 μM).^36^ As greater photodynamic inactivation was observed with vesicular rather than micellar suspensions, in this current work the length of the hydrophobic chain was increased to obtain vesicular structures, which can also be used as carriers for hydrophilic molecules owing to the aqueous lumen.^37^ To avoid the use of expensive and potentially toxic heavy metals, alternative organic photosensitisers were investigated.^38^ Phloxine B (PhB) is a hydrophilic xanthene dye approved for use as a colour additive in the US and Japan that is also known to act as a photosensitiser.^39,40^ As PhB does not absorb in the higher wavelength range of the visible region of the spectrum, it is a good candidate for superficial wound treatment.^41^ PhB has previously been incorporated into saponite-based films (PhB surface concentration ∼1.8 × 10^−6^ mmol cm^−2^) and was shown to provide an antibacterial effect against *S. aureus* with up to a 3 log_10_ reduction in CFU mL^−1^ after irradiation (green LEDs, 2.42 mW cm^−2^, 2.5 hours, 21.8 J cm^−2^).^42^ Polyurethane composites containing PhB afford a slow release of PhB. A 2 log_10_ reduction in CFU mL^−1^ was observed in the dark, attributed to the composite itself, with a further 2 log_10_ reduction after irradiation (green laser, *λ* = 532 nm, 100 mW, 120 s).^43^ However, encapsulation of PhB into polymersomes has not yet been reported. The aim of this study was to investigate the encapsulation of PhB into carriers composed of the biodegradable block copolymer PEG-*b*-PLA, and to investigate their potential to generate ^1^O_2_ and kill bacteria. This would enable highly concentrated PhB delivery at the target site.

In this work, a library of PEG-*b*-PLA block copolymers was synthesised and characterised with ^1^H-NMR spectroscopy and thermogravimetric analysis (TGA). The amphiphilic block copolymer was self-assembled into vesicles loaded with PhB, and characterised *via* Dynamic Light Scattering (DLS), Scanning Electron Microscopy (SEM) and Transmission Electron Microscopy (TEM). The PhB-loaded vesicles were then assessed for their ability to generate singlet oxygen using the sensor Singlet Oxygen Sensor Green (SOSG). Finally, incubation of the PhB-loaded vesicles with planktonic *S. aureus* and *P. aeruginosa* cultures, followed by irradiation for a short time with low-power green LEDs (4 mW cm^−2^, 15 minutes, 3.6 J cm^−2^) enabled determination of their antimicrobial efficiency. This system provides a rapid eradication of both clinically relevant wound infection pathogens with FDA-approved materials, highlighting a promising approach for combatting wound infections.

## 2. Experimental section

### 2.1. Materials

Poly(ethylene glycol) methyl ether (mPEG_114_, average number average molar mass (*M*_n_ = 5000 gmol^−1^)), l-lactide ((3S)-*cis*-3,6-dimethyl-1,4-dioxane-2,5-dione, 98%) and stannous octoate (tin(ii) 2-ethylhexanoate, 92.5–100%) were obtained from Sigma-Aldrich (Merck, Germany). Spectra/Por^®^ dialysis membranes (molecular weight cut-off 6–8 kDa) were purchased from Carl Roth GmbH (Germany). Phloxine B (2,4,5,7-tetrabromo-4,5,6,7-tetrachlorofluorescein disodium salt, dye content ≥80%) was purchased from Sigma-Aldrich (Merck, Germany). Milli-Q water was drawn from a Millipore Direct Q8 system with a resistivity of ≥18.2 MΩ cm (Millipore advantage A10 system, Schwalbach, with Millimark Express 40 filter, Merck, Germany).

All solvents were used as purchased: dichloromethane (DCM, 99.9%) (Fisher Scientific, Germany), tetrahydrofuran (THF, 100%) (VWR, Germany), diethyl ether (99.5%) (Thermos Scientific, Germany), methanol (99.9%) (Sigma-Aldrich, Merck, Germany) and chloroform-d (CDCl_3_, 99.8 atom%) (Deutero, Germany).

Acrylic cuvettes and transparent flat-bottom polystyrene 96-well plates were purchased from Sarstedt (Germany), and cell culture 96 black half-area μ-clear well plates and 384-well plates from Greiner Bio-One (Germany). Glass NMR tubes (Boro 400-5-7) were purchased from Deutero GmbH (Germany).

Lysogeny broth (LB medium, 10 g per L tryptone, 5 g per L yeast extract, 10 g per L NaCl, pH 7.0 ± 0.2) and LB agar (10 g per L tryptone, 5 g per L yeast extract, 10 g per L NaCl, 15 g per L agar–agar, pH 7.0 ± 0.2) were purchased from Carl Roth GmbH (Germany). Phosphate buffer solution (PBS, pH 7.4) was prepared by dissolving a PBS tablet (Sigma-Aldrich, Merck, Germany) in 200 mL Milli-Q water. Simulated thin wound exudate (STWE) was prepared by dissolving sodium chloride (NaCl, 0.8298%, Sigma-Aldrich, Merck, Germany) and calcium chloride (CaCl_2_, 0.0368%, AppliChem, Germany) in Milli-Q water. Singlet Oxygen Sensor Green (SOSG) was purchased from Thermo Fischer Scientific (Germany), and Tris buffer (100 mM, pH 7.4) from Millipore (MA, USA).

*Pseudomonas aeruginosa* lab strain ATCC 19660 (isolated from sepsis in Lima, Peru, and purchased from LGC Standard GmbH (Germany)) and *Staphylococcus aureus* lab strain RN4220 (ATCC 35556; originally derived from NCTC 8325-4 *via* UV and chemical mutagenesis, purchased from Leibniz-Institut DSMZ GmbH (Germany)) were used in this study. All bacteria were grown in LB medium at 37 °C.

### 2.2. Methods

#### 2.2.1. Synthesis of PEG-*b*-PLA

Prior to synthesis, all solid educts were dried overnight at reduced pressure (5 mbar), and all glassware was dried overnight at 60 °C. The synthesis was performed on the Schlenk line. After assembly of glassware, the Schlenk tubes were dried under vacuum with a heat gun, then flushed with argon after cooling. This was repeated three times. The adding of educts was performed under argon atmosphere.

The synthesis of PEG-*b*-PLA was adapted from Tücking *et al.*^44^ Molar ratios were adjusted according to desired PLA chain length. Briefly, with amounts corresponding to a nominal PLA chain length of 500, mPEG (100 μmol) was added to stannous octoate (0.1 mL) at 80 °C and stirred. After heating to 130 °C, l-lactide (25 mmol) was added and the mixture was stirred for 30 min. After cooling to room temperature, the white solid obtained was dissolved in dichloromethane (∼10 mL) and recrystallised in ice-cold diethylether (>10× volume of solvent). The precipitate was centrifuged (6000×*g*, 10 min), the supernatant discarded, and the precipitate redissolved in dichloromethane. This process was repeated a further 4 times, and the final white solid product was dried overnight (40 °C, 5 mbar).

#### 2.2.2. Characterisation of polymers

The polymers were characterised by ^1^H-NMR spectroscopy in CDCl_3_. Spectra were recorded on a Jeol ECZ 500 MHz spectrometer. The spectra were referenced to the residual proton signal of the solvent (*δ*(1H) = 7.26 ppm for CDCl_3_) and processed with the software MestReNova (version 6.0.2-5475). The observed chemical shifts (*δ*) are stated in ppm relative to the solvent signal, and *J* values are given in Hz.

##### 2.2.2.1. PEG-*b*-PLA_100_

*δ*_H_ (500 MHz; CDCl_3_): 7.26 (1H, s, C*H*Cl_3_), 5.16 (82H, q, ^3^*J* 7.1, CH_3_C*H*(C=O)O (PLA)), 3.64 (456H, s, OC*H*_2_C*H*_2_O (PEG)), 3.36 (3H, s, OC*H*_3_ (PEG-OMe)), 1.56 (267H, d, ^3^*J* 7.1, C*H*_3_CH(C=O)O (PLA)) (Fig. S1†).

##### 2.2.2.2. PEG-*b*-PLA_150_

*δ*_H_ (500 MHz; CDCl_3_): 7.26 (1H, s, C*H*Cl_3_), 5.16 (134H, q, ^3^*J* 7.1, CH_3_C*H*(C=O)O (PLA)), 3.64 (456H, s, OC*H*_2_C*H*_2_O (PEG)), 3.36 (3H, s, OC*H*_3_ (PEG-OMe)), 1.57 (429H, d, ^3^*J* 7.1, C*H*_3_CH(C=O)O (PLA)) (Fig. S1†).

##### 2.2.2.3. PEG-*b*-PLA_300_

*δ*_H_ (500 MHz; CDCl_3_): 7.26 (1H, s, C*H*Cl_3_), 5.16 (310H, q, ^3^*J* 7.1, CH_3_C*H*(C=O)O (PLA)), 3.64 (456H, s, OC*H*_2_C*H*_2_O (PEG)), 3.36 (3H, s, OC*H*_3_ (PEG-OMe)), 1.56 (952H, d, ^3^*J* 7.1, C*H*_3_CH(C=O)O (PLA)) (Fig. S1†).

##### 2.2.2.4. PEG-*b*-PLA_400_

*δ*_H_ (500 MHz; CDCl_3_): 7.26 (1H, s, C*H*Cl_3_), 5.16 (406H, q, ^3^*J* 7.1, CH_3_C*H*(C=O)O (PLA)), 3.64 (456H, s, OC*H*_2_C*H*_2_O (PEG)), 3.36 (3H, s, OC*H*_3_ (PEG-OMe)), 1.57 (1258H, d, ^3^*J* 7.1, C*H*_3_CH(C=O)O (PLA)) (Fig. S1†).

##### 2.2.2.5. PEG-*b*-PLA_500_

*δ*_H_ (500 MHz; CDCl_3_): 7.26 (1H, s, C*H*Cl_3_), 5.16 (483H, q, ^3^*J* 7.1, CH_3_C*H*(C=O)O (PLA)), 3.64 (456H, s, OC*H*_2_C*H*_2_O (PEG)), 3.36 (3H, s, OC*H*_3_ (PEG-OMe)), 1.57 (1707H, d, ^3^*J* 7.1, C*H*_3_CH(C=O)O (PLA)) (Fig. S2†).

The number of repeat units in the hydrophobic block was determined by calculating the ratio of the integral of the proton signals of the mPEG to the PLA block. The integral of the mPEG protons at 3.64 ppm was normalised to 456 protons, assuming a *M*_n_ of 5000 g mol^−1^. The integrals of the proton signals for the PLA protons were divided by the number of protons in 1 PEG repeat unit to calculate the number of PLA repeat units.

Thermogravimetric analysis (TGA) was performed on a TGA Q50 V6.7 Build 203 (Universal V4.4A, TA Instruments). The sample was dried overnight at 5 mbar, filled into a platinum crucible, placed in the autosampler of the device, and equilibrated at 50 °C under nitrogen atmosphere. After equilibration, the temperature was increased (50 °C min^−1^) to 500 °C. The gas was then changed to oxygen to oxidise remaining organic residues and the temperature was increased (50 °C min^−1^) from 500 to 850 °C.

#### 2.2.3. Encapsulation of Phloxine B

Self-assembled structures were prepared *via* the solvent shift method. To a stirring solution of polymer (1.00 mL, 3 wt% in THF), 1 mL of PhB solution (1 mM in water) was added at a rate of 0.5 mL min^−1^ with a syringe pump (AL-400, WPI, US). The milky suspension formed was stirred at room temperature, in the dark, for 16–18 hours, then 10 mL Milli-Q water was added. The suspension was dialysed for 7 days against >5 L Milli-Q water (MWCO 6–8 kDa) to remove residual polymer, THF and PhB, exchanging the surrounding water 3–4 times per day.

PhB concentration and encapsulation efficiency were determined *via* fluorescence spectroscopy. Emission spectra were recorded using a Varian Cary Eclipse spectrometer (Mulgrave, Victoria, Australia) at 25 °C. A calibration curve of PhB in water was created (*λ*_ex_/*λ*_em_ = 504/555 nm, 0–3.125 μM, *R*^2^ = 0.99) (Fig. S3†). The emission intensity of blank (water-filled) vesicles was subtracted from PhB-loaded vesicles, then PhB concentration inside the vesicle suspension (diluted to 5% v/v in Milli Q water) was determined by comparison with the calibration curve. This was also verified by comparison to the absorption calibration curve (0–50 μM, *R*^2^ = 0.99) (Fig. S4†). Encapsulation efficiencies were calculated as the ratio of the concentration of PhB in the final suspension relative to the initial PhB concentration.

#### 2.2.4. Characterisation of self-assembled structures

To determine average hydrodynamic diameter and size distributions, the vesicle suspensions were measured *via* Dynamic Light Scattering (DLS). Samples were diluted with Milli-Q water (10% v/v). Aliquots of 30 μL were measured in triplicate in a Wyatt Dynapro Plate Reader III (laser wavelength 818.3 nm, DLS detector angle 150°), with 5 acquisitions made per measurement (5 s per acquisition). Measurements were recorded at 25 °C. A Gauss fit (OriginPro 2018) was used to calculate the mean hydrodynamic diameter by number, and a mean and standard deviation of 3 independent measurements (3 samples) was calculated.

The samples were observed *via* Field Emission Scanning Electron Microscopy (FESEM, Zeiss Ultra 55cv, Zeiss, Oberkochen, Germany) by drop casting a diluted vesicle suspension (1% v/v in Milli Q water) onto silicon wafers pre-cleaned with chloroform, ethanol, water and UV-ozone. To generate a conductive surface, samples were sputtered with gold for 30 s, corresponding to a ∼5.0 ± 0.5 nm thin gold layer. FESEM measurements were collected using the Inlens secondary electron detector, with an operation voltage of 5 kV. The images were analysed using ImageJ (software version 1.53r).

Transmission Electron Microscopy (TEM, FEI Talos F200X) images of samples drop casted onto copper grids (mesh 400) with ultra-thin carbon film (<3 nm) supported by lacy-carbon-film (Plano GmbH, Germany) were recorded using an acceleration voltage of 80–200 kV. The images were analysed using ImageJ.

The structures were also observed *via* epifluorescence microscopy, using an Axiovert 135 microscope equipped with an AxioCamMRm and Zen 2.3 lite software and a filter set with *λ*_ex_ = 540–552 nm and *λ*_em_ > 590 nm (Carl Zeiss MicroImaging GmbH, Germany). The camera exposition time was 80 ms in brightfield, and 2000 ms with the filter set.

#### 2.2.5. Irradiation experiments

Irradiation was performed using a home-built device (Fig. S5†). A flat bottom polystyrene 96-well plate (Sarstedt, Germany) was half-filled with 48 green LED bulbs (3 V, 20 mA, type 15505, *λ*_max_ = 520 nm (Fig. S6†), 4 mW cm^−2^). This well plate was placed directly on top of the plate being irradiated. Unless stated otherwise, after a 15-minute incubation period, samples were irradiated thrice for 5 minutes, with 5 minutes in the dark (covered in aluminium foil) between each exposure. The irradiation was carried out at room temperature (22 °C). The radiant exposure was calculated with the following equation:Radiant exposure (J cm^−2^) = irradiance (W cm^−2^) × exposure time (s)

#### 2.2.6. Bacterial culture preparation

A sterile loop was used to streak glycerol stocks onto LB agar plates, which were incubated at 37 °C for 18 h. To prepare overnight bacteria cultures, a single colony was picked from an LB agar plate and used to inoculate 5 mL of LB medium, followed by incubation at 37 °C for 18 h while shaking at 200 rpm. Bacterial pellets were isolated by centrifugation of the overnight culture (5000×*g*, 5 min, 4 °C) and removal of the supernatant. The pellets were washed twice with STWE, and the optical density at 600 nm (OD_600_) adjusted in STWE to 0.6 (*P. aeruginosa*) or 1.2 (*S. aureus*), corresponding to ∼10^8^ CFU mL^−1^. This solution was used as prepared or further diluted 10× or 100× in STWE to obtain final working concentrations of ∼10^7^ and 10^6^ CFU mL^−1^.

#### 2.2.7. Detection of singlet oxygen production

The fluorescent probe singlet oxygen sensor green (SOSG) was used to detect ^1^O_2_.^45^ A 5 mM stock solution of SOSG was prepared by dissolving the contents of a vial (100 μg) in 33 μL methanol. Aliquots of 100 μM were prepared in Tris buffer and stored in the dark at −20 °C. To determine singlet oxygen production, 45 μL of sample was mixed with 45 μL of medium (Milli Q or bacterial culture in STWE) in a well of a black 96 well plate. Then, 10 μL of SOSG (100 μM in methanol) was added to reach a final SOSG concentration of 10 μM. Fluorescence emission intensity (*λ*_ex_ = 504 ± 5 nm, *λ*_em_ = 528 ± 5 nm) was measured (Tecan Safire (F129013), Tecan, Switzerland) after regular intervals of light irradiation. Each sample was measured in triplicate.

#### 2.2.8. Antimicrobial photodynamic therapy with planktonic bacteria

In a 96-well plate, 25 μL bacteria was incubated with 25 μL PhB-loaded vesicles or neat PhB solution (34 μM). The final volume in each well was a 2× dilution of both the treatment and the bacteria suspension. Each sample was tested in technical triplicate. The samples were incubated in the dark for 15 minutes at room temperature, then irradiation was performed. Non-irradiated samples were kept in the dark for the same time duration. To evaluate the aPDT effect, each technical replicate was serially diluted (10×) in PBS and 3 × 10 μL was plated for each dilution step on LB agar plates, resulting in a limit of detection of 34 CFU mL^−1^. Plates were incubated for 16 h at 37 °C, then bacteria colonies were counted. From this count, the log_10_ (CFU mL^−1^) and the log reduction factor (LRF) were calculated. For the LRF, the non-irradiated bacteria suspension was taken as the control. The arithmetic mean and standard deviation were calculated from 3 independently performed experiments (biological triplicate). Statistical significance was determined by one-way analysis of variance (ANOVA) and the Tukey multiple comparison test using OriginPro 2018. *p* values < 0.05 were considered statistically significant.

## 3. Results & discussion

**Scheme 1:**
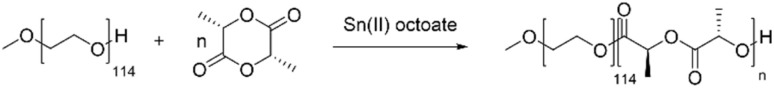
Ring-opening polymerisation of l-lactide with the macroinitiator mPEG_114_ and Sn(ii) octoate as catalyst.

PEG-*b*-PLA was synthesised *via* the ring-opening polymerisation of l-lactide with the macroinitiator mPEG, according to Scheme 1. This reaction was performed in the melt, and the molar ratio of monomer to macroinitiator was varied to obtain PEG-*b*-PLA copolymers with varying PLA chain lengths, with yields of 67–69% following reprecipitation.

The final products were characterised by ^1^H-NMR spectroscopy to determine the PLA block length. The ^1^H-NMR spectra (Fig. S1 and S2†) confirm the successful synthesis of the polymers, with characteristic proton signals for PEG and PLA blocks observed at 3.64 ppm (PEG), 1.56 ppm and 5.16 ppm (PLA). The ratios of the integrals of the PLA to PEG block proton signals were used to calculate the number average degree of polymerisation (*M*_n_), and the hydrophobic block length (Table 1). The calculated chain length was close to the expected chain length (86–98% expected value).

**Table 1:** Number average molecular weight (*M*_n_) and corresponding hydrophobic block length of the synthesised polymers according to ^1^H-NMR

Nominal PLA chain length, *x*	*M*_n_/kg mol^−1^		Hydrophobic block length/number of repeat units	
	Expected	Observed	Expected	Observed
100	12.2	11.6	100	92
150	15.8	15.3	150	143
300	26.6	26.2	300	294
400	33.8	32.9	400	389
500	41.0	40.1	500	488

Amphiphilic block copolymers can self-assemble into various morphologies depending on the composition of the initial polymer. The resulting morphology is dependent on the packing parameter, *p*, which is calculated by division of the volume of the hydrophobic chains by the hydrophilic headgroup area and hydrophobic tail length.^16^ Micelles are formed when *p* ≤ ⅓, and possess a hydrophobic core composed of hydrophobic tails, while the hydrophilic head group is in contact with the surrounding water. Vesicles are formed when 1/2 ≤ *p* ≤ 1, and are composed of a polymeric bilayer with hydrophilic head groups in contact with both the exterior and interior solvent, creating an aqueous lumen in which hydrophilic molecules can be encapsulated. In this work, the aim was to create a high local concentration of PhB by loading it into a carrier. Hence, the encapsulation of the hydrophilic PhB molecule was achieved *via* formation of vesicular structures, which provide a large lumen for higher loading capacity. To ensure vesicles were obtained in the self-assembly of PEG-*b*-PLA, the polymer with the longest hydrophobic PLA chain (and hence the largest *p*) was chosen for further studies.

As previously reported, gel permeation chromatography (GPC) was unsuccessful with this system due to the inherent differences in solubility of the two blocks.^36^ Hence, TGA was used to provide a weight average degree of polymerisation for the polymer with expected PLA chain length of 500. The TGA trace (Fig. S7†) shows distinctive mass losses at the decomposition temperatures of PEG (330–400 °C) and PLA (150–250 °C).^46^ The ratio of the residual weight lost at the characteristic decomposition temperatures was used to calculate the PLA block length and was in good agreement with the ^1^H-NMR data (*M*_w_: 40.3 kg mol^−1^, PLA block length: 490 compared to *M*_n_: 40.1 kg mol^−1^, PLA block length: 488 from ^1^H-NMR). Therefore, from hereon in, this polymer will be referred to as PEG_114_-*b*-PLA_488_.

The self-assembly of PEG_114_-*b*-PLA_488_ was achieved *via* nanoprecipitation. Here, the amphiphilic block copolymers are dissolved in a common solvent for both blocks, in this case THF. The addition of water, a non-solvent for the hydrophobic PLA chains, results in the formation of nanoassemblies *via* microphase separation.^14,47^ To encapsulate PhB, a 1 mM solution in Milli-Q water was used as the non-solvent.

**Fig. 1:**
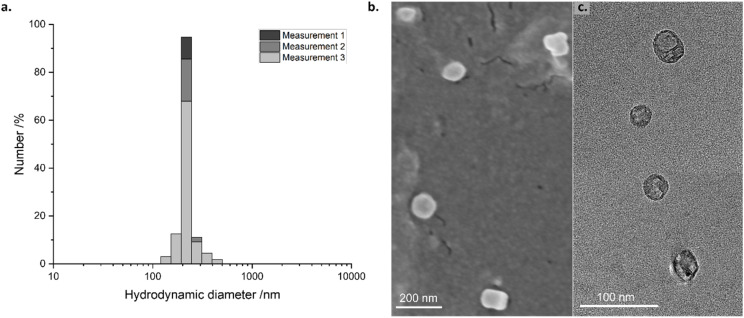
(a) DLS particle size distribution by number, (b) FESEM and (c) TEM images of PhB-loaded PEG_114_-*b*-PLA_488_ vesicles (1% dilution in Milli-Q water).

The resulting suspensions were characterised by DLS, FESEM and TEM (Fig. 1). The distributions by number of the suspensions indicated an average hydrodynamic diameter of 230 ± 7 nm for PhB-loaded PEG_114_-*b*-PLA_488_ assemblies (Fig. 1a). The FESEM and TEM images (Fig. 1b and c) exhibit spherical features that are likely vesicles, according to the report of Reverchon *et al.*, who studied very similar PEG-*b*-PLA polymers.^48^ These round features possess diameters (101 ± 16 nm) that are consistent with the DLS data. These images also confirm that the spherical shapes are largely retained upon drying, indicating structural stability of the assemblies upon drying.

**Fig. 2:**
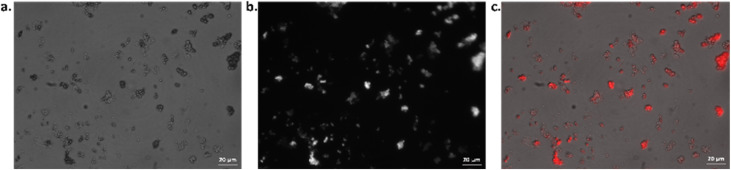
Epifluorescence microscopy image of PhB-loaded PEG-*b*-PLA vesicles in (a) bright field, (b) emission resulting from a filter set with *λ*_ex_ = 540–552 nm and *λ*_em_ > 590 nm and (c) bright field and emission images are merged to localise the PhB, with pseudo colouring of the fluorescence emission. Scale bars: 20 μm.

To confirm that PhB is encapsulated, fluorescence microscopy images were acquired. By overlaying the bright field and emission images, it is possible to visualise clusters of vesicles which contain the fluorescent PhB (Fig. 2). These fluorescent spots are not visible in water-loaded vesicles, confirming they are due to the presence of PhB (Fig. S8†).

To estimate the concentration of PhB in the final vesicle suspension, the fluorescence emission of the vesicle suspension (diluted to 5% v/v in Milli Q water) was measured and compared to the calibration curve of neat PhB in water (Fig. S9†). The concentration of PhB in the vesicle suspension was estimated to be 34 ± 1.6 μM, corresponding to an encapsulation efficiency of 3.4 ± 0.2% (Fig. S9b†). Attempts to quantify loading with UV-vis spectroscopy proved difficult as the scattering from the polymer shell in the vesicle suspension prevented any estimation of PhB concentration (Fig. S10†). Hence the absorption spectra were used only to confirm the presence of the dye in the suspension.

**Fig. 3:**
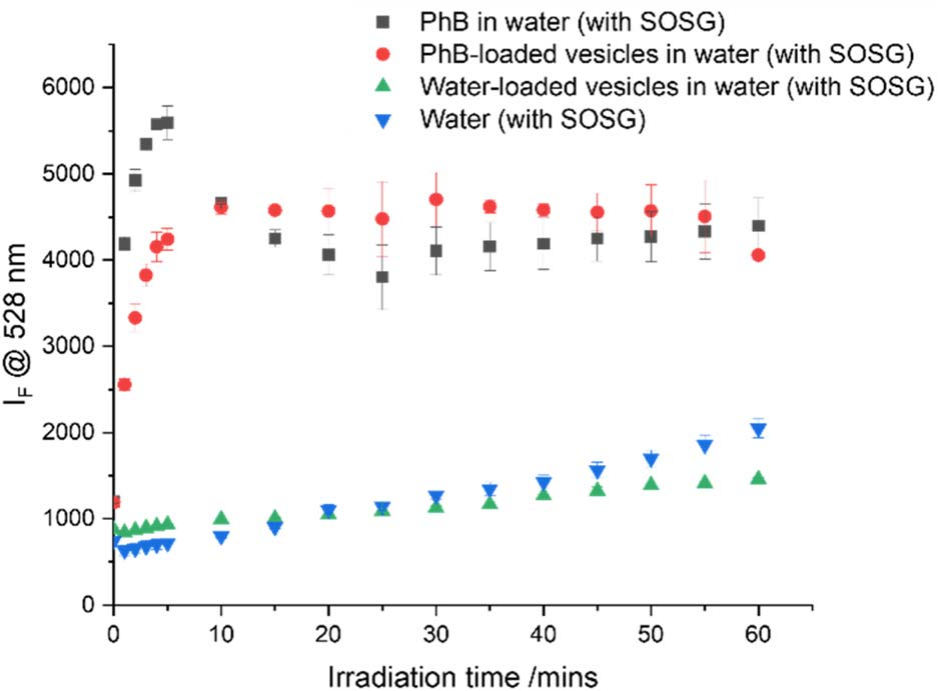
SOSG probe signal to detect ^1^O_2_ production of neat PhB, PhB-loaded PEG_114_-*b*-PLA_488_ vesicle suspensions and water with increasing irradiation times (*λ* = 520 nm, 4 mW cm^−2^). The error bars represent the standard deviation (*n* = 3).

PhB is known to generate reactive oxygen species primarily *via* a Type II energy transfer from the triplet excited state to molecular oxygen, affording singlet oxygen, ^1^O_2_.^18,42^ Singlet Oxygen Sensor Green (SOSG) was therefore used as a probe molecule to confirm the production of ^1^O_2_. This non-fluorescent fluorescein-based probe reacts with ^1^O_2_ to produce an SOSG-endoperoxide which exhibits green fluorescence (Fig. S11†).^49^ The conversion of SOSG to SOSG-endoperoxide in the presence of PhB and PhB-loaded vesicles upon irradiation was monitored by measuring the fluorescence emission intensity at 528 nm. The impact of increasing irradiation time of PhB and PhB-loaded vesicles on ^1^O_2_ production was investigated (Fig. 3).

As controls, water and water-loaded vesicles were also irradiated in the presence of SOSG. Irradiation of SOSG (10 μM in Milli Q water) in the absence of a photosensitiser resulted in an increase in the fluorescence emission intensity over time, suggesting the fluorescein moiety itself is excited by the green light, forming ^1^O_2_ and self-converting to SOSG-endoperoxide.^50^ It has been reported that both the SOSG and the SOSG endoperoxide derivative can sensitise ^3^O_2_, generating more ^1^O_2_ and therefore further increasing SOSG fluorescence.^25,51^ For these reasons, SOSG was not used to quantify ^1^O_2_ production, only to qualitatively detect it. Irradiation of water-loaded vesicles showed a similar trend to SOSG alone, confirming the lack of ^1^O_2_ production from the polymer itself. However, the fluorescence intensity of PhB and PhB-loaded vesicles was up to 4-fold higher than the signal from the SOSG itself. This indicates that the irradiation of both neat dye and dye loaded vesicles generates ^1^O_2,_ converting SOSG to SOSG-endoperoxide. Nevertheless, it can be observed in Fig. 3 that upon increasing irradiation times beyond 15 minutes, there is no further apparent ^1^O_2_ production, as has been observed with other systems.^52^ This saturation could be consistent with the consumption of all methylanthracene moieties or depletion of oxygen in the local environment.^24^ It could also be due to the visible light-induced degradation of PhB (Fig. S12†), resulting in fewer molecules available to sensitise molecular oxygen.^53^

**Fig. 4:**
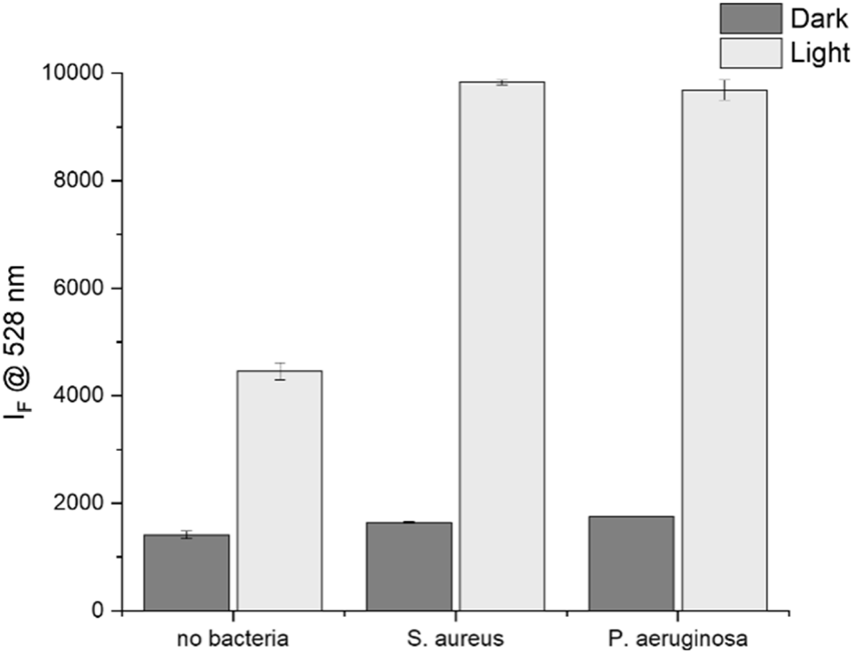
SOSG probe signal to detect ^1^O_2_ production of PhB loaded PEG_114_-*b*-PLA_488_ vesicle suspensions kept in the dark (dark) or irradiated for 15 minutes (*λ* = 520 nm, 4 mW cm^−2^) (light). Samples were first incubated for 15 minutes with an equal volume of either STWE (no bacteria), *S. aureus* (10^6^ CFU mL^−1^ in STWE) or *P. aeruginosa* (10^6^ CFU mL^−1^ in STWE).

To confirm the production of ^1^O_2_ in the presence of bacteria, the vesicle suspensions were pre-incubated for 15 minutes with *S. aureus* and *P. aeruginosa* in STWE. This industry standard medium, also referred to as solution A, is a good approximation to mimic a wound exudate.^54,55^ An increase in fluorescence emission at 528 nm, indicating an increase in ^1^O_2_ production, was detected upon irradiation of PEG_114_-*b*-PLA_488_ vesicle suspensions in the presence of both *S. aureus* and *P. aeruginosa* and SOSG (Fig. 4 and S13†). In comparison to the signal in the absence of bacteria, the emission after 15 minutes of irradiation in the presence of both *S. aureus* and *P. aeruginosa* is >2× higher. Such an increase has been observed before.^36^ This could be an indication that the PS is interacting with the bacteria, increasing the local concentration of ^1^O_2_ and therefore enhancing the SOSG signal. In addition, upon irradiation of PhB, the bacteria are under oxidative stress, triggering antioxidant reactions which generate reactive intermediates which may non-specifically activate SOSG.^56^ Moreover, the permeabilisation of bacterial membranes upon ^1^O_2_ treatment may facilitate SOSG staining, as has been observed in *E. coli*, creating environments with concentrated SOSG.^57^

**Fig. 5:**
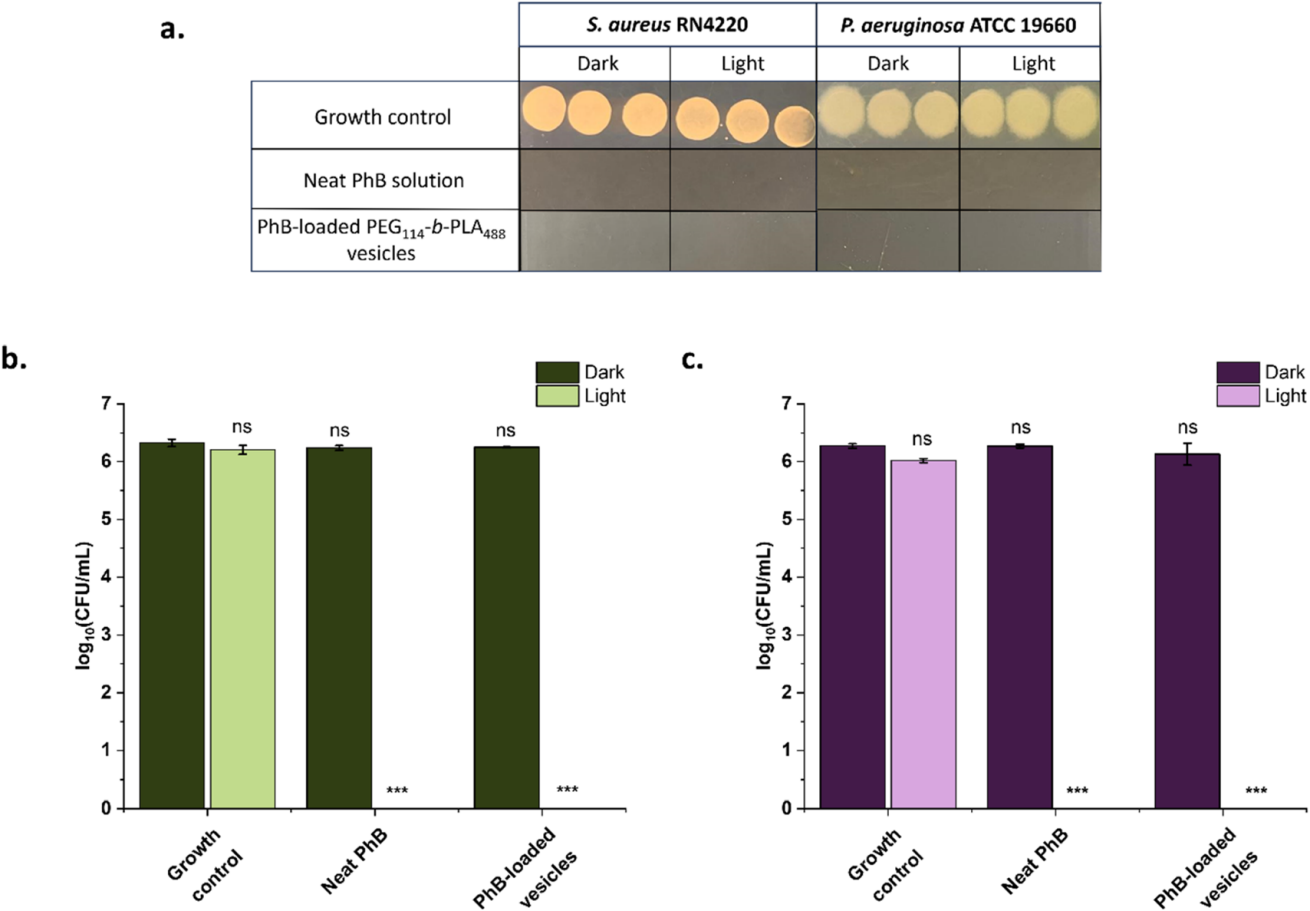
(a) Exemplary photographs of bacterial colonies visible after 18 hours growth at 37 °C following irradiation of samples for 15 minutes (light) or dark incubation (dark) (inoculum 10^6^ CFU mL^−1^). Antimicrobial effectiveness of neat PhB and PhB-loaded PEG_114_-*b*-PLA_488_ against (b) *S. aureus* RN4220 and (c) *P. aeruginosa* ATCC 19660. The samples were irradiated for 15 min (*λ* = 520 nm, 4 mW cm^−2^) (light) or incubated in the dark for 15 min (dark). The error bars correspond to the standard deviation of biological replicas (*n* = 3). Asterisks represent significant difference from non-irradiated growth control (**: *p* < 0.01, ***: *p* < 0.001, ns: no significant difference).

The PhB-loaded vesicle suspensions were used as produced in antibacterial tests against *S. aureus* lab strain RN4220 and *P. aeruginosa* lab strain ATCC 19660. These strains were chosen as they are often reported to be resistant to antibiotic treatment; RN4220 is a methicillin-resistant *S. aureus* (MRSA) lab strain, a key target of aPDT treatment. Both bacteria were used at a starting concentration of ∼10^6^ CFU mL^−1^, a concentration which is associated with increased infection risk in wound exudates, and the experiment was performed in the clinically relevant STWE.^58^ Both neat PhB and PhB-loaded vesicles were incubated in equal volumes with the bacteria and the CFU mL^−1^ after 15 minutes of irradiation (radiant exposure 3.6 J cm^−2^) or dark incubation were determined. Exposure to light irradiation in the absence of vesicles, or in the presence of water-loaded vesicles, had no effect on cultures of *S. aureus* or *P. aeruginosa* (Fig. 5 and S14†). Likewise, both neat PhB and PhB-loaded PEG-*b*-PLA vesicle suspensions caused no reduction in CFU mL^−1^ of *S. aureus* or *P. aeruginosa* in the dark. However, upon irradiation of neat PhB and PhB-loaded vesicles, there were no countable *S. aureus* or *P. aeruginosa* colonies observed (Fig. 5a). This represents a log reduction factor (LRF) of >4.8 for *S. aureus*, and >4.7 for *P. aeruginosa*.

The eradication of *P. aeruginosa* in these experiments was unexpected, as neat Phloxine B is commonly reported to be active only against Gram-positive bacteria.^59^ Gram-negative bacteria, including *P. aeruginosa*, are generally more difficult to kill due to their cell wall structure. Combination treatment of aPDT with chelating agents, antibiotics or antimicrobial peptides to permeabilise the cell wall and facilitate PS uptake has been proposed.^60–62^ The use of neat PhB to photodynamically inactivate *P. aeruginosa* has not yet been reported. In general, Gram-negative bacteria are often resistant to neutral and anionic PSs.^62^ Antimicrobial activity of Phloxine B has been achieved against Gram-negative bacteria in the presence of EDTA, a chelating agent which binds to cations holding the lipopolysaccharides (LPS) of the cell wall in place, which increases cell permeability and enables dye penetration.^59^ Possibly the Ca^2+^ ions in STWE in this experiment are permeabilising the LPS, a process known and used for decades in DNA transformation of cells.^63^ Moreover, the presence of Cl^−^ counterions in the STWE may be providing a synergistic effect, as they can combine with the singlet oxygen to produce the longer-lived hypochlorite, ClO^−^, which can also have an antimicrobial effect.^64,65^

**Fig. 6:**
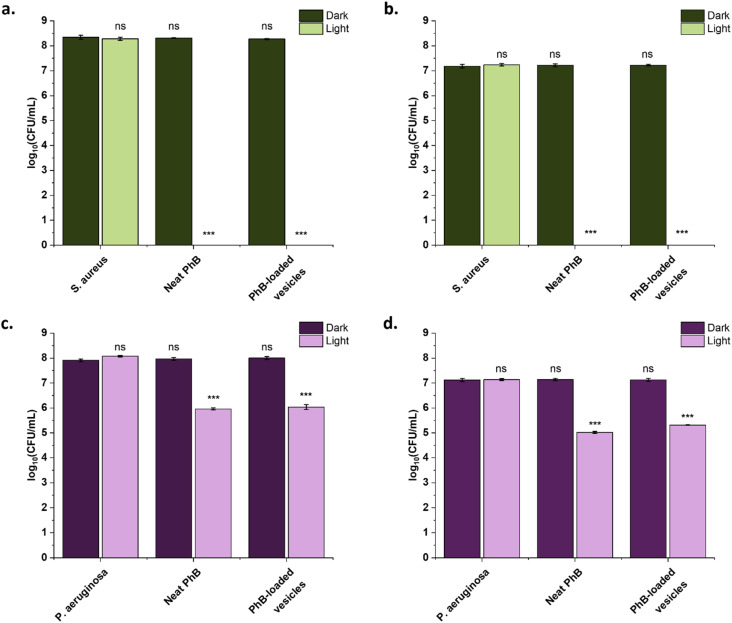
Antimicrobial effectiveness of neat PhB and PhB-loaded PEG_114_-*b*-PLA_488_ against (a and b) *S. aureus* RN4220 and (c and d) *P. aeruginosa* ATCC 19660. Bacteria at different starting concentrations were tested: (a and c) ∼10^8^ CFU mL^−1^, (b and d) ∼10^7^ CFU mL^−1^. The samples were irradiated for 15 min (*λ* = 520 nm, 4 mW cm^−2^) (light) or incubated in the dark for 15 min (dark). The error bars correspond to the standard deviation of biological replicas (*n* = 3). Asterisks represent significant difference from non-irradiated growth control (**: *p* < 0.01, ***: *p* < 0.001, ns: no significant difference).

To investigate the extent of the antibacterial activity of the developed PhB-loaded vesicle systems, the experiment was repeated with increasing bacteria concentrations, 10^7^ CFU mL^−1^ and 10^8^ CFU mL^−1^. Increasing the *S. aureus* concentration did not limit the antibacterial activity of neat or encapsulated PhB upon irradiation (Fig. 6a and b), with still no colonies visible after irradiation (Fig. S15 and S16†). These data now provide a LRF of >6.8 (accounting for a LOD of 1.5 log_10_ CFU mL^−1^), indicating that >99.9999% bacteria are eradicated after just 15 minutes. From Fig. 6c and d, it is evident that with higher *P. aeruginosa* concentrations, the ability of this concentration of PhB to eradicate the bacteria is limited, with a maximum LRF of 2, compared to the LRF of >4.7 when the inoculum is ∼10^6^ CFU mL^−1^. This highlights the importance of early detection of *P. aeruginosa* infections for utilisation of PhB treatment. However, it is likely that longer irradiation times or more concentrated vesicle suspensions would enable killing of higher inoculum concentrations.

It is advantageous to eradicate both *S. aureus* and *P. aeruginosa* as polymicrobial wound infection is common; 27% of wounds in one study were diagnosed with a polymicrobial infection, most commonly *S. aureus* and *P. aeruginosa*.^66^ The susceptibility and antibiotic resistance patterns of different bacterial species and strains differs. Hence, multiple antibiotics would be required to treat one wound. With this approach, both Gram-positive and Gram-negative species could be eradicated with one treatment, using biocompatible polymer carriers which localise the PS. The lack of reported microbial resistance to aPDT is likely due to the short interval between PS administration and light irradiation, in which time there is no time to select for resistant strains.^67^ In addition, the success with a low-cost, low-power, non-coherent LED array enables treatment over a large area due to the wide illumination area relative to a laser light source, without causing damage to healthy tissue.^19^ Further work to optimise the concentration of Phloxine B inside the carriers with regards to the irradiation time will provide an “optimal” system with a low active concentration with the minimum treatment time.

## 4. Conclusions

The amphiphilic block copolymer PEG-*b*-PLA was synthesised with varying hydrophobic block lengths. PEG_114_-*b*-PLA_488_ was self-assembled into vesicles with a typical diameter of 100 nm. These were loaded with the photosensitiser Phloxine B. Irradiation of these vesicles with a simple LED set-up completely eradicates both planktonic *P. aeruginosa* and *S. aureus* with a log reduction factor of >6.8 and >4.8, respectively, depending on starting bacteria concentration. By immobilising this system into a support matrix, successful application as a wound dressing is hypothesised. A combinatorial diagnostic-therapeutic aPDT approach would improve the clinical applicability. If combined with a reporter dye, upon detection of a wound infection, irradiation would efficiently kill both *P. aeruginosa* and *S. aureus*, the two most common wound infection pathogens.

## Data availability

The data supporting this article have been included as part of the ESI† and will be made available on the open access depository Zenodo.

## Author contributions

Nicola Cusick: methodology, investigation, formal analysis, validation, visualisation, data curation, writing – original draft; Holger Schönherr: conceptualisation, supervision, funding acquisition, project administration, resources, writing – review & editing.

## Conflicts of interest

There are no conflicts to declare.

## Supplementary Material

RA-015-D5RA02238J-s001
