# Supplementary material for: Phloxine B-loaded polymersomes enable eradication of Pseudomonas aeruginosa and Staphylococcus aureus in antimicrobial photodynamic therapy
Source: RSC Adv. 2025 Jun 4;15(24):18815–25. doi: 10.1039/d5ra02238j (PMC12135823; doi:10.1039/d5ra02238j)
Supplement: RA-015-D5RA02238J-s001 [file RA-015-D5RA02238J-s001.pdf]

Supplementary Information (SI) for RSC Advances.
This journal is © The Royal Society of Chemistry 2025

Supporting Information

Phloxine B-loaded polymersomes enable photodynamic eradication of *Pseudomonas aeruginosa* and *Staphylococcus aureus* in antimicrobial photodynamic therapy

Nicola Cusick, Holger Schönherr*

Physical Chemistry I & Research Center of Micro- and Nanochemistry and (Bio)Technology (Cμ), Department of Chemistry and Biology, School of Science and Technology, University of Siegen, 57076 Siegen, Germany

*corresponding author: schoenherr@chemie.uni-siegen.de

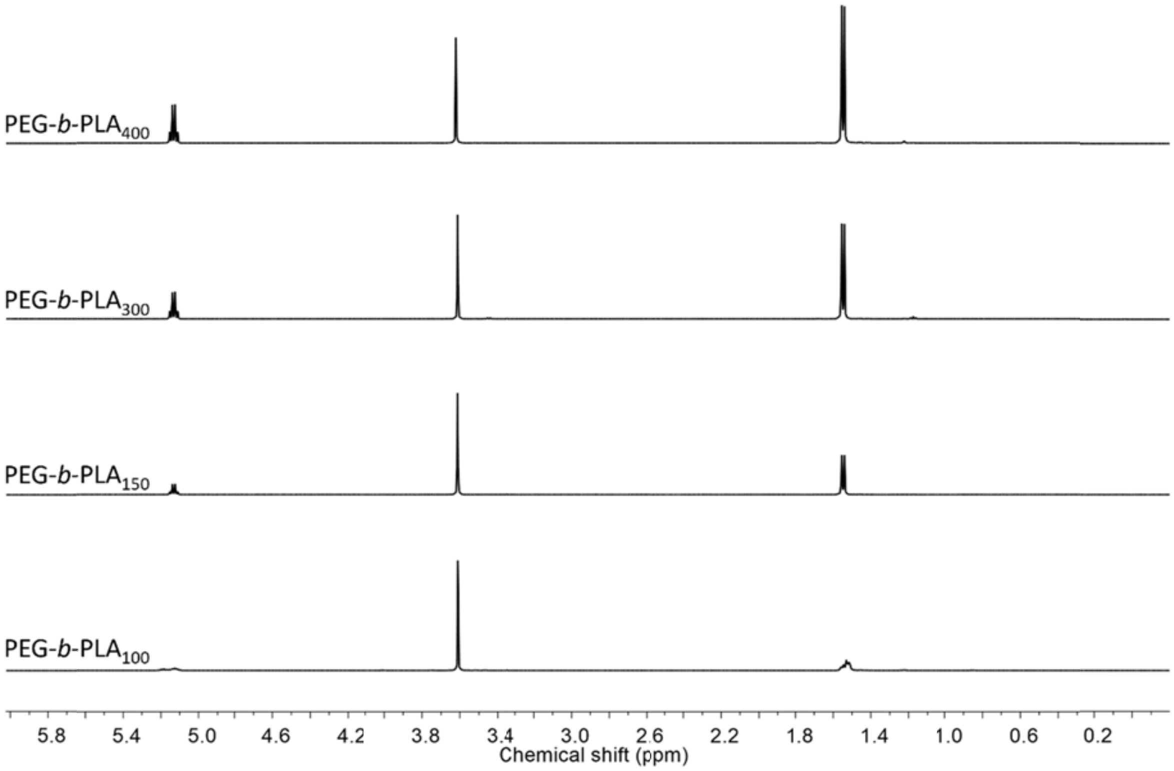

Figure S1 ^{1}H-NMR spectra of the synthesised PEG-b-PLA polymers with nominal PLA block length 100-400, measured in $CDCl_3$.

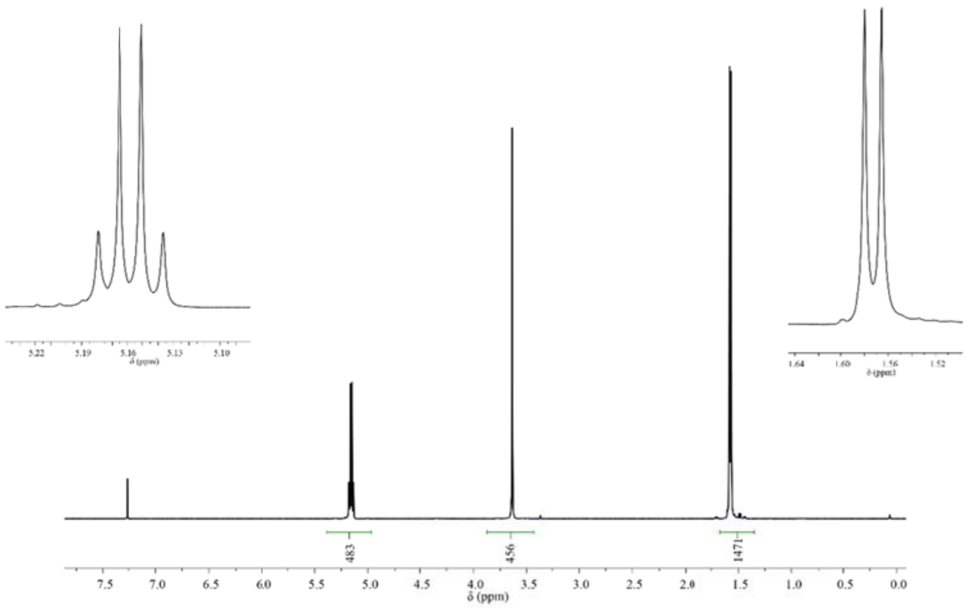

Figure S2: ^{1}H-NMR spectrum of the synthesised PEG-*b*-PLA_{500} in $CDCl_3$.

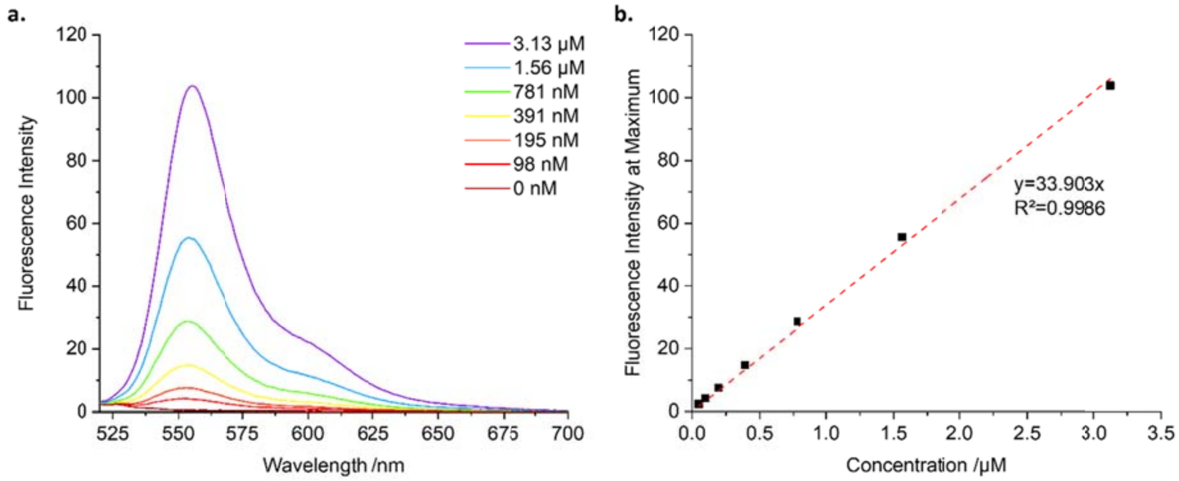

Figure S3: a) Fluorescence emission spectra of PhB in Milli-Q water (λ_{ex}=504 nm) and b) resulting calibration curve of fluorescence intensity at the maximum emission wavelength as a function of PhB concentration.

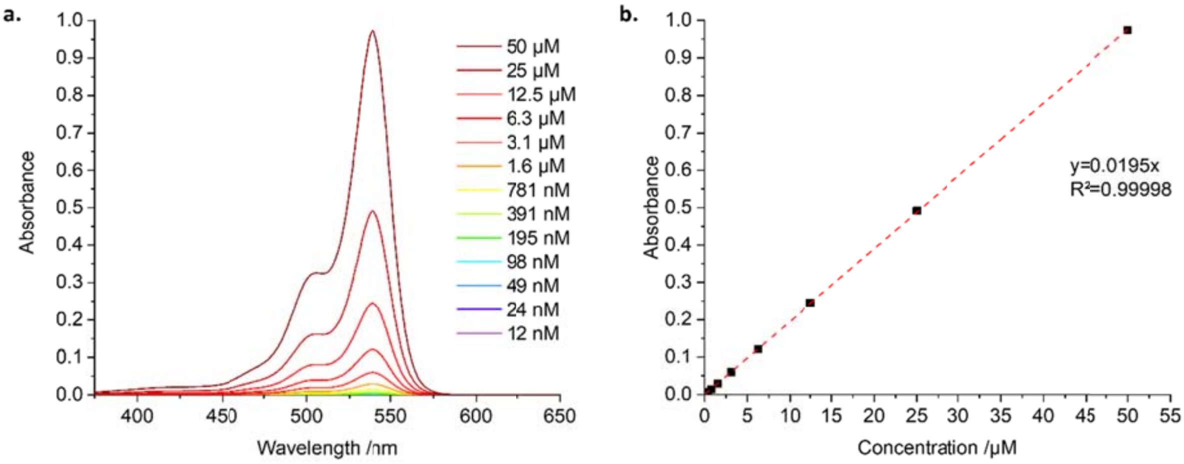

Figure S4: a) Absorption spectra of PhB in Milli-Q water and b) associated calibration curve of absorbance at maximum absorption wavelength vs PhB concentration.

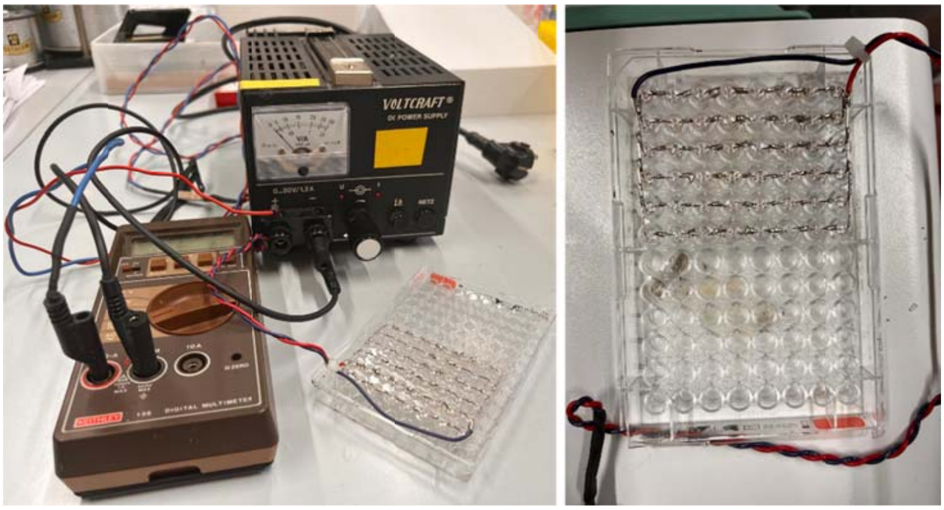

Figure S5. Home built irradiation setup, consisting of a 96 well plate containing 48 green LEDs, connected to a DC power supply via a digital multimeter.

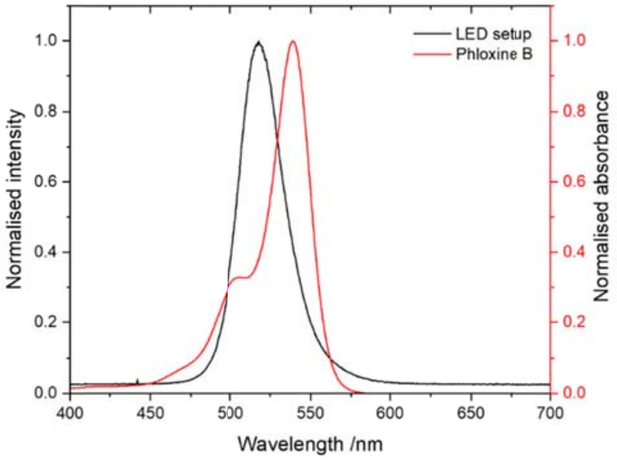

Figure S6: Emission wavelength of the green LED setup overlayed with the normalised absorption spectrum of Phloxine B

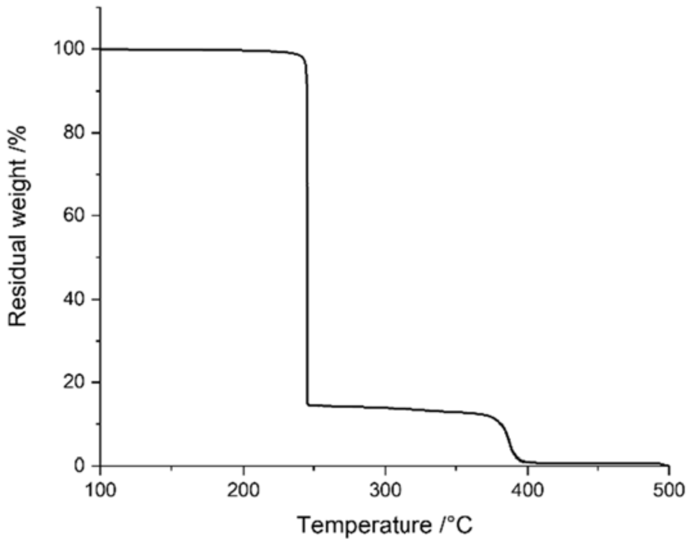

Figure S7: TGA trace of the synthesised PEG-*b*-PLA block copolymer with nominal PLA block length of 500 under N_2 atmosphere (50 °C/min).

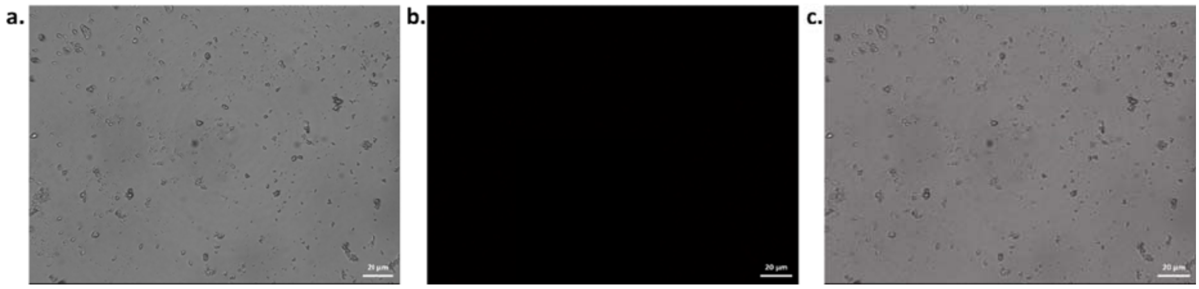

Figure S8 Epifluorescence microscopy image of water-loaded PEG-*b*-PLA vesicles in a) bright field, b) emission resulting from a filter set with λ_{ex} = 540–552 nm and λ_{em} > 590 nm and c) bright field and emission images are merged. Scale bars: 20 µm.

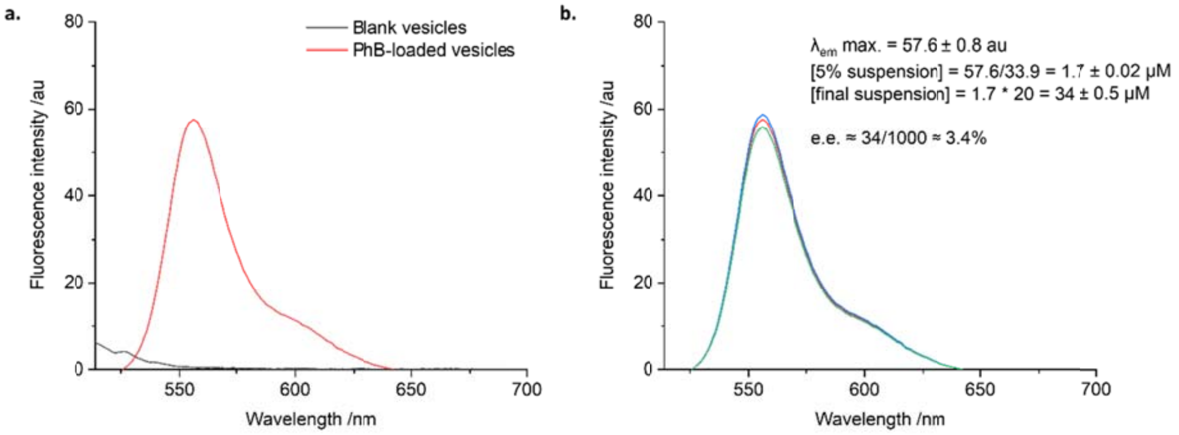

Figure S9: Fluorescence emission spectra of a) blank (water-filled) and PhB-loaded PEG_{114}-*b*-PLA_{488} vesicle suspensions and b) 3 separately prepared PhB-loaded PEG_{114}-*b*-PLA_{488} vesicle suspensions. All suspensions were measured at 5% v/v concentration in Milli Q water.

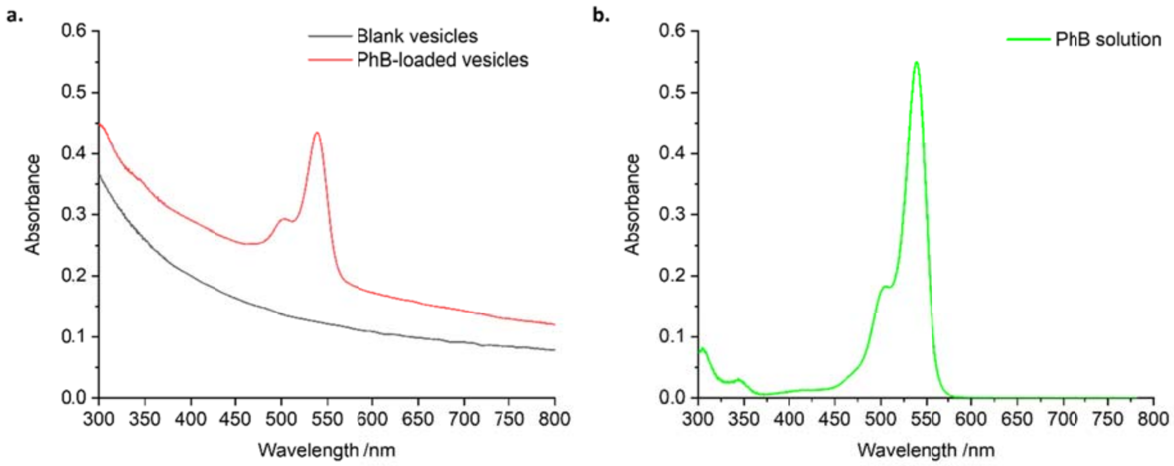

Figure S10: Absorption spectra of a) blank (water-filled) and PhB-loaded PEG$_{114}$-*b*-PLA$_{488}$ vesicle suspensions measured at 5% v/v concentration in Milli Q water, and b) neat PhB solution (10 µM in water).

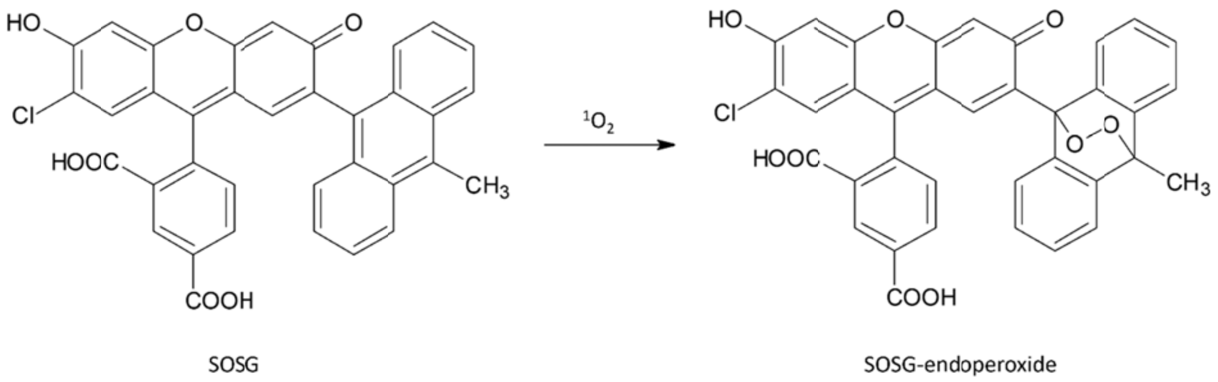

Figure S11: The conversion of the SOSG probe into its endoperoxide form in the presence of singlet oxygen.

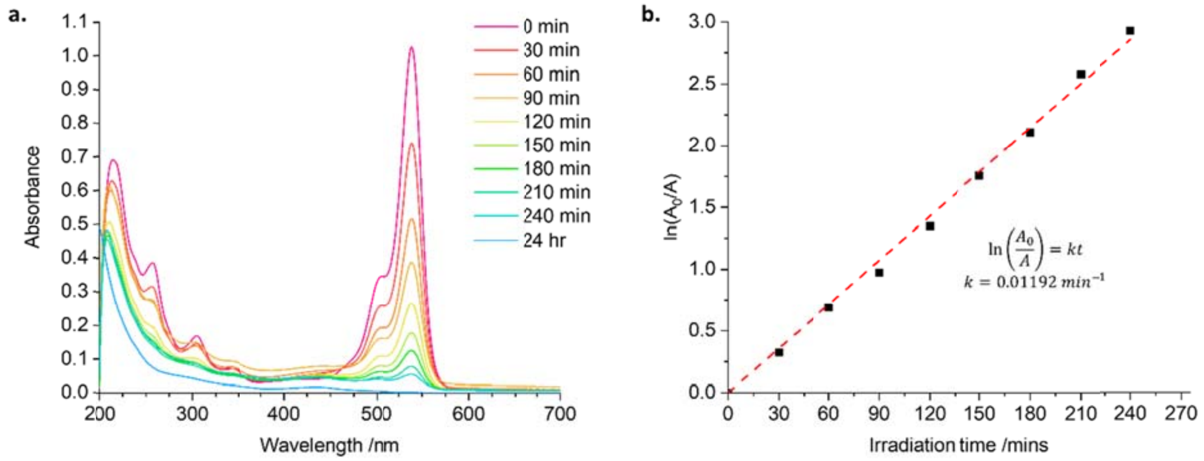

Figure S12: (a) Absorption spectra of Phloxine B in Milli-Q water with increasing irradiation time. (b) Relative absorption at 539 nm vs irradiation time, resulting in an apparent first order rate constant (k) of photodegradation of 0.01192 min^{-1}. In this experiment, irradiation of the sample was performed in a quartz cuvette on a 575 W halogen lamp (Ed. Liesegang type 643, Germany).

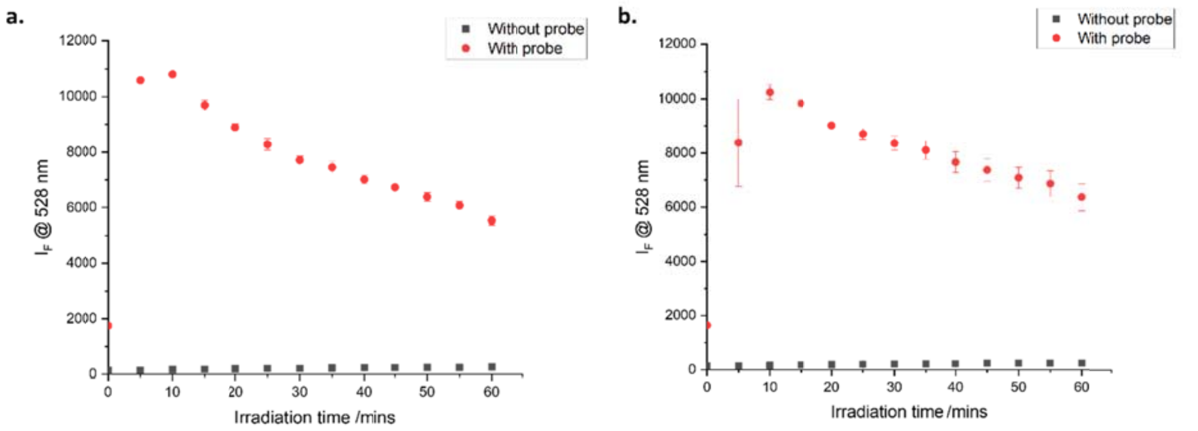

Figure S13: SOSG probe signal with increasing irradiation time (λ=520 nm, 4 mW/cm^2) of PhB-loaded PEG-b-PLA vesicles with a. *P. aeruginosa* and b. *S. aureus* (both 10^6 CFU/mL in STWE) with Tris buffer (without probe) or SOSG (with probe).

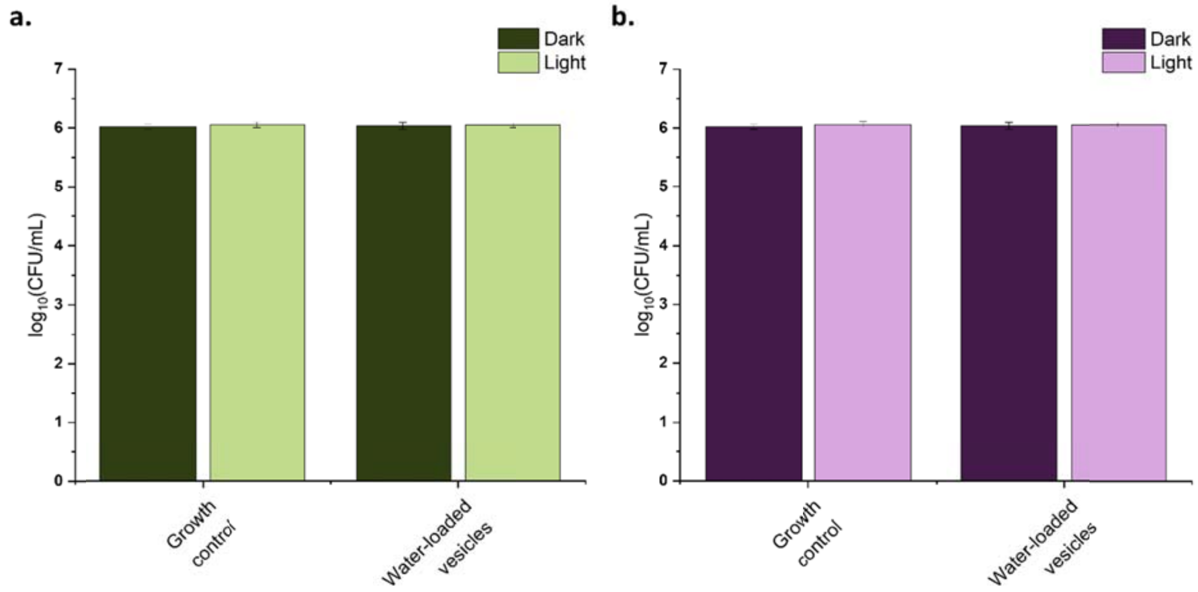

Figure S14 Antimicrobial effectiveness of water-loaded PEG_{114}-b-PLA_{488} vesicles against a) *S. aureus* RN4220 and b) *P. aeruginosa* ATCC 19660 with a starting concentration of 10^6 CFU/mL. The samples were irradiated for 15 min (λ=520 nm, 4 mW/cm^2) (Light) or incubated in the dark for 15 min (Dark). The error bars correspond to the standard deviation of biological replicas (n = 3).

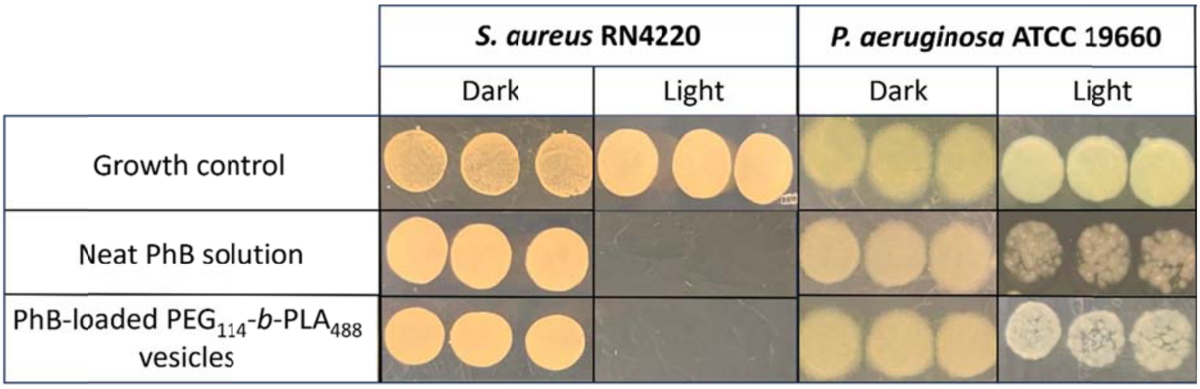

Figure S15. Exemplary photographs of bacterial colonies visible after 18 hours growth at 37°C following irradiation of samples for 15 minutes (Light) or dark incubation (Dark) (inoculum 10^7 CFU/mL).

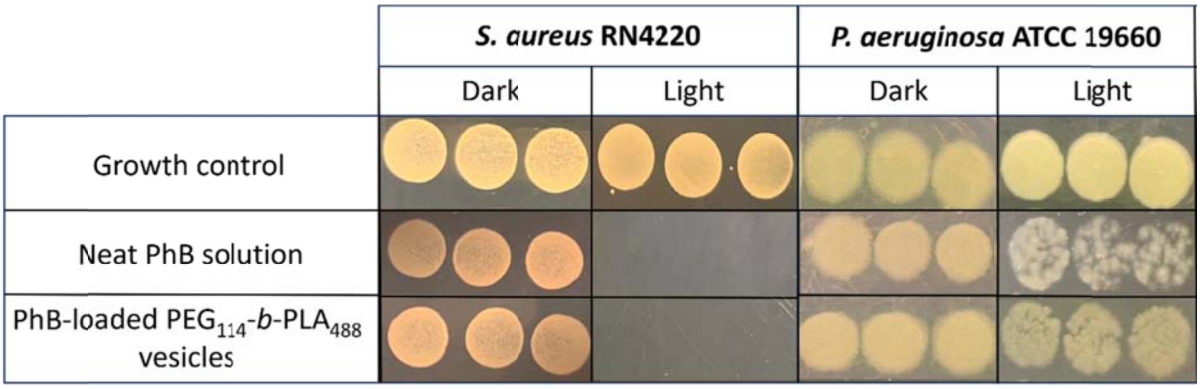

Figure S16. Exemplary photographs of bacterial colonies visible after 18 hours growth at 37°C following irradiation of samples for 15 minutes (Light) or dark incubation (Dark) (inoculum 10^8 CFU/mL).
